# *In Silico* Screening of Marine Compounds as an Emerging and Promising Approach against Estrogen Receptor Alpha-Positive Breast Cancer

**DOI:** 10.1155/2021/9734279

**Published:** 2021-12-17

**Authors:** Abdulwahab Alamri, Abdur Rauf, Anees Ahmed Khalil, Adel Alghamdi, Ahmed Alafnan, Abdulrahman Alshammari, Farhan Alshammari, Jonaid Ahmed Malik, Sirajudheen Anwar

**Affiliations:** ^1^Department of Pharmacology and Toxicology, College of Pharmacy, University of Hail, Hail, Saudi Arabia; ^2^Department of Chemistry, University of Swabi, Anbar, Swabi, Khyber Pakhtunkhwa (KP), Pakistan; ^3^University Institute of Diet and Nutritional Sciences, Faculty of Allied Health Sciences, The University of Lahore, Pakistan; ^4^Department of Pharmaceutical Chemistry, Faculty of Clinical Pharmacy, Al Baha University, Al Baha, Saudi Arabia; ^5^Department of Pharmacology and Toxicology, College of Pharmacy, King Saud University, Riyadh, Saudi Arabia; ^6^Department of Pharmaceutics, College of Pharmacy, University of Hail, Hail, Saudi Arabia; ^7^Department of Pharmacology and Toxicology, National Institute of Pharmaceutical Education and Research, Guwahati, India

## Abstract

Presently, the majority of breast tumors are estrogen receptor (ER) positive. Breast cancer (BC) is defined by uncontrolled cell proliferation (CP) in breast tissue. BCs are caused by the overexpression of genes that promote CP in breast cells. The discovery of effective inhibitors is an excellent chemopreventive method. Our *in silico* approach analysis offers a pharmacoinformatics methodology for identifying lead molecules targeting cochaperone HSP90 and the epidermal growth factor receptors (EGFR) and human epidermal growth factor receptor 2 (HER2)/neu receptor. BC has been associated with the high expression of these targets. The use of drug-likeness filters aided in determining the therapeutic properties of possible lead compounds. In this study, docking-based virtual screening (VS) was performed. Database of about 450 cancer marine compounds was used. The X-ray-assisted structure of ER*α* with 4-OHT (PDB code: 3ERT) was chosen for 4-OHT. A docking-based virtual screening was performed on the dataset supplied using the molecular operating environment (MOE) dock application. The binding energy (BE) and explanation of the protein inhibitor interaction (PII) are crucial findings for future both in terms of dry or wet lab research. The GBVI/WAS binding-free energy assessment (in kcal/mol) scores were used to grade the compounds. Compounds with a BE of less than -9.500 kcal/mol were deemed to be the most effective inhibitors. For further analysis, the top seven structurally diverse scaffolds were selected. Seven marine compounds exhibited the best docking score, which validates them to be potent anti-BC compounds. These compounds' bioactive potential and prospective drug-likeness profile make them promising leads for further experimental research.

## 1. Introduction

Breast cancer (BC) is responsible for around half million deaths and 1.2 million new cases every year. It is the primary cause of female mortality (cancer accounts for 23 percent of all cancer cases and 14 percent of cancer deaths) [1]. In 2012, mortality rates in women due to this cancer were reported to be 15.5 percent in developed economies (189,000 deaths) and 12.7 percent in emerging economies (269,000 deaths), respectively [2]. According to the Saudi Cancer Registry of the King Faisal Specialist Hospital and Research Centre (KFSH), around 930 new BC patients are diagnosed each year in Saudi Arabia. BC was the most frequently newly diagnosed cancer among Saudi women in 2010, contributing to 27.4 percent of all malignancies diagnosed [3].

Estrogen levels are associated with the genesis of osteoporosis and, breast and uterine cancers. The estrogen receptor (ER) is found in the endometrium, BC, and ovarian stromal cells, and also the hypothalamic, and promotes cell proliferation (CP) [4]. Tamoxifen, a selective ER modulator, is a widely used antiestrogen adjuvant medication for ER-positive (ER+) premenopausal women selective estrogen receptor modulators (SERM). Tamoxifen's active metabolite is 4-hydroxytamoxifen (4-OHT) [5]. Tamoxifen is also commonly used to treat postmenopausal women with ER+ malignancies. In ER+ BC cells, it acts as an ER antagonist, blocking the ER signalling pathway. As a result, tamoxifen medication dramatically lowers the chance of recurrence of BC. In addition, the tamoxifen-bound ER complex prevents estrogen from turning on genes, hence preventing the estrogenic actions that cause cancer cell growth [6].

Tamoxifen has a clear advantage in the treatment of BC, but it also has significant side effects. Because of its agonistic impact in the uterus, for instance, the threat of endometrial cancer (EC) and hyperplasia increases 1.5- to 6.9-fold [7] following cumulative and long-term use [8] because of its agonistic effect in the uterus. Furthermore, the risk of EC increased considerably in overweight postmenopausal females. To complicate matters further, many ER+ women, regardless of ER levels, showed intrinsic resistance to hormonal treatments. As a result, alternative therapies are required.

BC is among the most perilous and often diagnosed cancers in women [9]. BC, with a prevalence of 21.8 percent, is a widespread malignancy among Saudi women [10]. According to the most recent cancer-related mortality study, breast cancer is the leading cause of mortality among Saudi women. Resistance to therapeutic agents is a significant issue in the treatment of cancer disorders, and it is thought to impair the efficacy of selective therapies as well as the prognosis of cancer patients [11]. BC is a heterogeneous illness on the molecular level, with molecular features such as activation of human epidermal growth factor receptor 2 (HER2), activation of hormone receptors like ER and progesterone (PR), and/or breast cancer gene (BRCA) mutations [12].

BC, defined as uncontrolled CP, causes a hard, pain-free lump in the breast tissue, most commonly in the milk ducts or lobules that supply milk [13, 14]. The most popular method for classifying BCs is based on the state of three distinct cell surface receptors: the ER, PR, and EGF (epidermal growth factor receptor) HER2/neu receptor [15]. Many biological problems have been solved using *in silico* techniques [16, 17], resulting in novel inhibitors against a wide range of diseases [15].

Marine pharmacology is a modern area that investigates the marine ecosystem in pursuit of possible medicinal drugs. Marine natural products (MNPs) are untapped resources with potential pharmacological properties. The harsh environmental conditions and competition within the biological systems make marine flora and fauna produce structurally distinct metabolites. To date, a significant number of MNPs have been identified as potential anticancer drugs. Furthermore, these MNPs play a vital role in inhibiting human tumor cells under laboratory conditions and in cancer clinical trials. To mention a few, glembatumumab vedotin derived from *Dolabella auricularia*-associated *Symploca* sp. is currently being tested for advanced or metastatic breast cancer in a phase I/II clinical trial [18]. Keeping in this mind, this study was aimed at exploring the potential of MNPs for the potential anti-BC drugs. The MarinLit database was used to extract the compounds for the study.

## 2. Materials and Methods

### 2.1. Protein Preparation

The study's key treatment targets for BC were ER-alpha. In addition, the 3DS of the following BC target protein (PDB code: 3ERT) was obtained from the protein data bank.

### 2.2. Dataset Preparation

The compounds were selected based on their reported activity against BC both in *in vitro* and *in vivo* studies. Thirteen one (31) compounds from the MarinLit database (RSC) and 176 compounds from Natural Product Updates (RSC) were used to analyze against the BC targets. The Ligand.mdb database of compounds was built from a SMILES format. Our previously disclosed methods were applied for ligand preparation, enzyme downloading, energy minimization, 3D protonation, and binding site determination. The MOE Builder tool was utilized to create the ligand structures. The compound database Ligand.mdb was made. The compounds were then energy-reduced up to 0.001 Gradient using the MMFF94X force field.

### 2.3. Docking-Based VS

Docking experiments were carried out using the MOE 2016.08. The MOE window was opened to view the enzyme structure. Water molecules were eliminated (if present). All atoms were 3D protonated in an implicit solubilized environment at 300 K temperature, pH = 7, and salt concentration of 0.1. The entire structure was energy minimized using the MMFF94X force field, and all chemicals were docked into the binding sites of the produced enzymes. Default docking settings were defined, and ten alternative conformations were constructed for each chemical. MOE ligand interaction module was utilized to evaluate low binding energy ligand enzyme complexes, while the Discovery Studio visualizer was employed for 3D interaction plots.

### 2.4. Drug Likeness Evaluation

To determine whether the active compounds had the potential to be developed as medication, we used Lipinski's “Rule of Five” [19] to predict oral bioavailability using the Molinspiration WebME editor 1.16 (http://www.molinspiration.com). The large percentage of orally administered medications have an MW of less than 500, an average log*P* of less than 5, five or fewer HB donation sites, and ten or fewer HB acceptor sites. Furthermore, bioavailability was determined using the TPSA analysis (http://www.molinspiration.com).

## 3. Results and Discussion

### 3.1. *In Silico* Docking Results

BC is a prevalent malignancy and has become the second leading cause of cancer death among women globally. Several medications for the treatment of breast cancer have been licensed by the US Food and Drug Administration. However, these treatments are costly and can have a variety of side effects. Patients frequently report fatigue, headaches, musculoskeletal problems, blood clots, lymphedema, fertility problems, loss of memory, and other side effects, which become necessary to seek alternative medicines from marine sources [20]. Some of these challenges can be addressed with new anticancer agents derived from marine sources. Several well-known marine compounds have been discovered [18]. However, only a few marine compounds and derivatives have been licensed for commercial usage, while many are now undergoing preclinical and clinical testing [18]. The current study is aimed at identifying alternative compounds from natural origin against BC target ER*α*. Today, the majority of breast tumors are ER*α*-positive. Chemotherapy is less effective in ER*α*-positive BC than in ER*α*-negative disease [21]. The binding energy and explanation of the PII are significant findings for future experimental and theoretical research.

SBVS on the database of about 450 cancer marine compounds was performed using MOE docking suite. The X-ray-derived structure of ER*α* in complex with 4-OHT (PDB code: 3ERT) was selected for VS.

### 3.2. Validation of Docking Protocol

Before the docking-based VS of the dataset, we performed a comprehensive validation of MOE docking protocol. For this purpose, cocrystallized ligand 4-OHT was extracted and redocked into the active site of 3ERT, and RMSD was calculated. For a prediction of ligand-target conformations, an RMSD cut-off value less than 2 Å is considered good [22]. Starting with the Triangle Matcher as placement stage algorithm and London dG scoring function, and GBVI/WSAdG final scoring function, we tried the alpha triangle (placement stage), while two other scoring functions, ASE and affinity dG, were also attempted for docking validation. Best performance in terms of 133 computed RMSD value, conformation, position, and pose (orientation) was obtained with Triangle Matcher and London dG scoring function.

### 3.3. Structure-Based Virtual Screening

**Figure 1:**
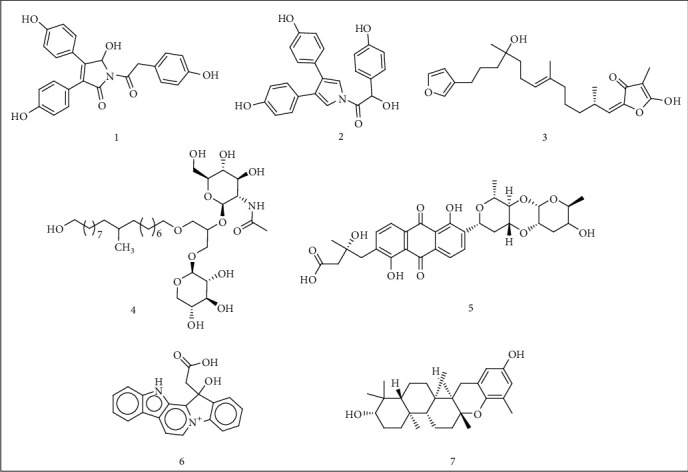
Structures of Top 7 structurally diverse scaffolds from SBVS experiment.

After docking protocol validation, SBVS of the dataset of compounds was carried out. The compounds were ranked by the scores by the GBVI/WAS binding free energy calculation (in kcal/mol). Compounds with binding energy values less than -9.500 kcal/mol were considered best for inhibition. Top 7 structurally diverse scaffolds were selected for further analysis (Figure 1).

5-Hydroxyneolamellarin B (1) was found to demonstrate the best docking conformation with the BE value of -10.2389 kcal/mol followed by 7-hydroxylamellarin A (2), 8-hydroxyisovariabilin (3), myrmekioside E-1 (4), vineomycin E (5), homofascaplysate A (6), and isoepitaondiol (7) (Figure 1). Computed BE values and RMSD refine (the root mean square deviation between the pose before refinement and the pose after refinement) are shown in Table 1.

Next, we analyzed the binding orientation of the identified seven compounds into the binding site of 3ERT. ER*α* possesses six functional domains A-F. Among them, three main functional domains are A/B domain, C domain, E domain, i.e., N-terminus, DNA binding domain, and LBD, respectively. Analysis of the 3ERT revealed that the key amino acid residues' lining active sites are Met343, Leu346, Thr347, Ala350, Asp351, Glu353, Trp383. Arp394, Glu419, Gly420, Met421, Gly521, and Leu525. Tamoxifen, a marketed ER*α* antagonist binds with Arg394 and thus inhibits and blocks the ER function.

**Figure 2:**
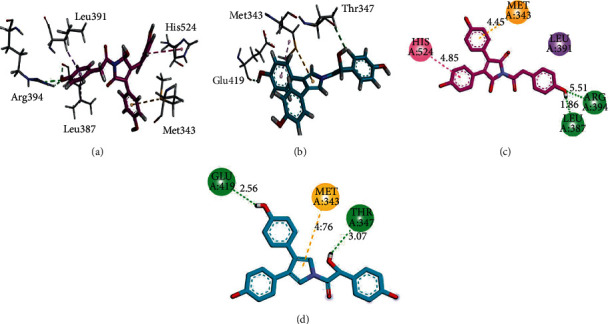
3D (a, b) and 2D (c, d) interaction plots of (a) 5-hydroxyneolamellarin B (1) and (b) 7-hydroxylamellarin A (2), into the binding site of 3ERT.

**Figure 3:**
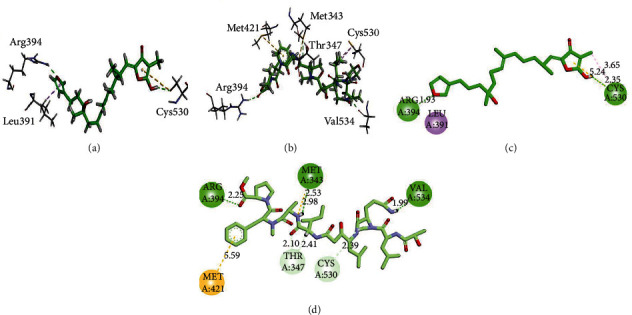
3D (a, b) and 2D (c, d) interaction plots of (a) 8-hydroxyisovariabilin (3) and 5 and (b) 7 myrmekioside E-1 (4) into the binding site of 3ERT.

**Figure 4:**
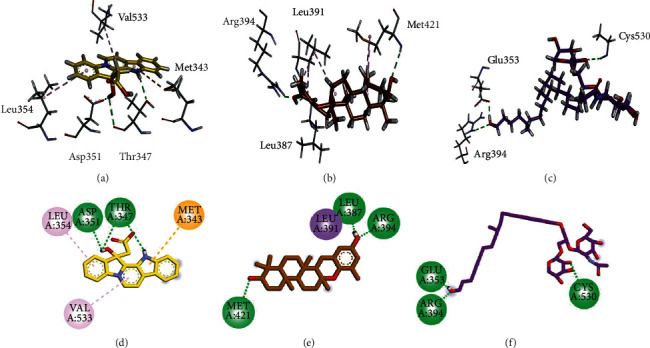
3D (a–c) and 2D (d–f) interaction plots of (a) vineomycin E (5) (b) homofascaplysate A (6) and isoepitaondiol (7) into the binding site of 3ERT.

Three/two-dimensional (3D/2D) binding interaction plots of the identified compounds are shown in Figures 2–4. The important interacting amino acid residues showing HB and hydrophobic interactions are listed in Table 1. Structures shown in Figure 1 revealed that all the identified compounds contain hydroxyl groups. These hydroxyl groups establish HB interactions with important residues Thr347, Glu353, Arg394, and Leu387. Phenyl rings form *π* − *π* stacking interaction (His524) and *π* − *σ* (Leu391) types of interactions. Met343 forms *π*-sulfur interactions.

### 3.4. *In Silico* Pharmacokinetic Prediction

Using online server AdmetSAR, we predicted the pharmacokinetic properties of the identified compounds. All the compounds, except myrmekioside E-1, showed excellent human intestinal absorption. Similarly, all compounds, except myrmekioside E-1 and vineomycin E, showed blood-brain barrier penetration. All the identified compounds were found noncarcinogen. Findings of in silico pharmacokinetic properties of identified compounds are listed in Table 2.

## 4. Conclusion

BC is one of the most common cancers in women worldwide. Computational techniques have been broadly used in drug discovery and the finding of multitargeted inhibitors of numerous upregulated proteins in BC. This research identifies five multitargeted drugs with strong BEs against the most prevalent target proteins involved in BC. Following *in vitro* and *in vivo* testing, these virtual hits with excellent PK and PD features may be considered for early therapeutic development against BC. The seven marine compounds exhibited the best docking score, which valid them to be potent anti-BC compounds. These compounds' bioactive potential and prospective drug-likeness profile make them promising leads for further experimental research. The chemicals investigated show that they could be employed as pharmaceuticals or as functional food additives with a promising role in creating medicines and nutritional supplements. Development of new functional ingredients/foods was mechanistically proven efficacy through *in vivo* screening, lead optimization, and bioavailability.

## Figures and Tables

**Table 1:** BEs, RMSD, and key interacting amino acid residues (H binding and hydrophobic binding).

No.	Binding energy (kcal/mol)	RMSD (Å)	Interacting residues	
			HB interactions	Hydrophobic interactions
1	-10.2389	0.95	Arg394, Leu87	His524 (*π* − *π*), Met343(*π*-S), Leu391 (*π* − *σ*)
2	-9.8315	1.33	Thr347, Glu419	Met343 (*π* − S)
3	-9.5103	0.79	Arg394, Cys530	Leu391 (*π* − *σ*)
4	-10.5086	1.075	Met343, Arg394, Val534	Met343 (*π*-S), Met421 (*π*-S)
5	-9.8805	1.33	Thr347, Asp351	Met343 (*π*-S)
6	-9.5830	0.92	Leu387, Arg394, Met421	Leu391 (*π* − *σ*)
7	-9.5440	1.146	Glu353, Arg394, Cys530	—

**Table 2:** *In silico* pharmacokinetic prediction of identified compounds.

Compounds	Human intestinal absorption	Blood-brain barrier	Carcinogenicity (binary)
5-Hydroxyneolamellarin B	0.9804	0.9675	No
7-Hydroxylamellarin A	0.9675	0.9688	No
8-Hydroxyisovariabilin	0.9475	0.9562	No
Myrmekioside E-1	-0.9349	-0.5458	No
Vineomycin E	0.9245	-0.4254	No
Homofascaplysate A	0.6659	0.9618	No
Isoepitaondiol	0.9936	0.9189	No

## Data Availability

The docking data used to support the findings of this study are available from the corresponding author.

## Abbreviations

3DS: 3-Dimensional structure
4-OHT: 4-Hydroxytamoxifen
BE: Binding energy
BC: Breast cancer
BRCA: Breast cancer gene
EGF: Epidermal growth factor
CP: Cell proliferation
HB: Hydrogen bond
HER2: Human epidermal growth factor receptor 2
EC: Endometrial cancer
ER: Estrogen receptor
EGFR: Epidermal growth factor receptors
LBD: Ligand binding domain
MW: Molecular weight
MOE: Molecular operating environment
PR: Progesterone receptor
PII: Protein inhibitor interactions
RMSD: Root square mean deviation
TSA: Topological surface area
SBVS: Structure-based virtual screening
VS: Virtual screening.
